# Potential Deep Brain Stimulation Targets for the Management of Refractory Hypertension

**DOI:** 10.3389/fnins.2019.00093

**Published:** 2019-02-25

**Authors:** Raleigh Ems, Anisha Garg, Thomas A. Ostergard, Jonathan P. Miller

**Affiliations:** Department of Neurological Surgery, Neurological Institute, University Hospitals Cleveland Medical Center, Cleveland, OH, United States

**Keywords:** deep brain stimulation, periaqueductal gray, hypertension, subthalamic nucleus, rostral subcallosal neocortex, Brodmann area 25, insular cortex, functional neurosurgery

## Abstract

Hypertension is the single greatest contributor to human disease and mortality affecting over 75 million people in the United States alone. Hypertension is defined according to the American College of Cardiology as systolic blood pressure (SBP) greater than 120 mm Hg and diastolic blood pressure (DBP) above 80 mm Hg measured on two separate occasions. While there are multiple medication classes available for blood pressure control, fewer than 50% of hypertensive patients maintain appropriate control. In fact, 0.5% of patients are refractory to medical treatment which is defined as uncontrolled blood pressure despite treatment with five classes of antihypertensive agents. With new guidelines to define hypertension that will increase the incidence of hypertension world-wide, the prevalence of refractory hypertension is expected to increase. Thus, investigation into alternative methods of blood pressure control will be crucial to reduce comorbidities such as higher risk of myocardial infarction, cardiovascular accident, aneurysm formation, heart failure, coronary artery disease, end stage renal disease, arrhythmia, left ventricular hypertrophy, intracerebral hemorrhage, hypertensive enchaphelopathy, hypertensive retinopathy, glomerulosclerosis, limb loss due to arterial occlusion, and sudden death. Recently, studies demonstrated efficacious treatment of neurological diseases with deep brain stimulation (DBS) for Tourette’s, depression, intermittent explosive disorder, epilepsy, chronic pain, and headache as these diseases have defined neurophysiology with anatomical targets. Currently, clinical applications of DBS is limited to neurological conditions as such conditions have well-defined neurophysiology and anatomy. However, rapidly expanding knowledge about neuroanatomical controls of systemic conditions such as hypertension are expanding the possibilities for DBS neuromodulation. Within the central autonomic network (CAN), multiple regions play a role in homeostasis and blood pressure control that could be DBS targets. While the best defined autonomic target is the ventrolateral periaqueductal gray matter, other targets including the subcallosal neocortex, subthalamic nucleus (STN), posterior hypothalamus, rostrocaudal cingulate gyrus, orbitofrontal gyrus, and insular cortex are being further characterized as potential targets. This review aims to summarize the current knowledge regarding neurologic contribution to the pathophysiology of hypertension, delineate the complex interactions between neuroanatomic structures involved in blood pressure homeostasis, and then discuss the potential for using DBS as a treatment for refractory hypertension.

## Introduction

Hypertension affects 32.6% of the US adult population and is one of the greatest risk factors for cardiovascular disease (Elijovich et al., 2016). The 2017 guidelines by the American College of Cardiology define hypertension as systolic blood pressure (SBP) above 120 mm Hg and diastolic blood pressure (DBP) above 80 mm Hg measured on two occasions, with stage 1 hypertension defined as SBP of 130–139 mm Hg or DBP of 80–89 mm Hg, and stage 2 hypertension as SBP greater than 140 mm Hg and DBP greater than 90 mm Hg (Whelton et al., 2017).

Despite multiple treatment options, fewer than 50% of hypertensive patients achieve appropriate blood pressure control with medication and up to 0.5% of patients are refractory to medical treatment (Howard et al., 2006; Calhoun et al., 2014). Refractory hypertension is defined as uncontrolled blood pressure in patients taking more than five classes of antihypertensive agents including a mineralocorticoid receptor (MR) antagonist as well as a thiazide-like diuretic that has a long duration of action (Dudenbostel et al., 2015, 2017). Resistant hypertension is distinct from refractory hypertension and is defined as an elevated blood pressure despite using three or more medications (as opposed to five) (Armario et al., 2017), including a diuretic (Dudenbostel et al., 2017). Furthermore, refractory hypertension is due to increased sympathetic outflow, whereas resistant hypertension is characterized by hypervolemia (Dudenbostel et al., 2017), and this has important implications for treatment strategies. The prevalence of hypertensive patients with resistant hypertension is estimated to be between 10 and 20% (Dudenbostel et al., 2017) and of refractory hypertension has been estimated to be 2.7% of patients with resistant hypertension (Dudenbostel et al., 2017). The new guidelines redefining SBP and DBP for hypertension are expected to increase the percentage of adults in the United States diagnosed with hypertension from 32 to 46% (Flack et al., 2018). Accordingly, the prevalence of refractory hypertension is also expected to increase.

Patients with resistant hypertension have a higher risk of myocardial infarction, cardiovascular accident, aneurysm formation, heart failure, coronary artery disease, end stage renal disease, arrhythmia, left ventricular hypertrophy, intracerebral hemorrhage, hypertensive enchaphelopathy, hypertensive retinopathy, glomerulosclerosis, limb loss due to arterial occlusion, and sudden death (Dudenbostel et al., 2017). Additionally, most patients with hypertension have additional co-morbidities such as diabetes, high cholesterol, obesity, and metabolic syndrome that increase morbidity and mortality. Compared to non-resistant hypertensives, patients with resistant hypertension are more likely to have chronic kidney disease, congestive heart failure, or ischemic heart disease as comorbid conditions (Ambrosius et al., 2014; SPRINT Research Group et al., 2015). While resistant hypertension is better described, cardiovascular outcomes from refractory HTN have not been quantified by longitudinal assessment, but studies indicate that cardiovascular risk is increased with refractory hypertension compared to resistant hypertension (Dudenbostel et al., 2017). Patient demographics also vary between resistant and refractory HTN, as those with refractory hypertension are more frequently female and a younger age (Dudenbostel et al., 2017).

Recent clinical applications of deep brain stimulation (DBS) are mainly in neurological diseases including Tourette’s (Shahed et al., 2007; Maciunas et al., 2012; Schrock et al., 2014; Smeets et al., 2018), depression (Lozano et al., 2008; Schlaepfer et al., 2008; Riva-Posse et al., 2014), intermittent explosive disorder (Giordano et al., 2016; Rizzi and Marras, 2017), epilepsy (Krishna et al., 2016; Lehtimaki et al., 2016), chronic pain (Boccard et al., 2015; Russo and Sheth, 2015), and headache (Altinay et al., 2015; Miller et al., 2016) (Deuschl et al., 2006; Weaver et al., 2009; Williams et al., 2010; Gabriëls and Nuttin, 2012; Ostergard and Miller, 2014). Specifically, DBS is an established treatment for movement disorders such as Parkinson’s Disease, and there are suggestions that other disorders might respond as well (Schuepbach et al., 2013). DBS consists of an array of electrodes placed into complex neuroanatomical structures and attached to an implantable pulse generator with adjustable settings (Hariz, 2014). The precise mechanism by which DBS controls neuronal activity is not fully understood, but the effects on neuronal circuitry have been well characterized (Johnson et al., 2013). Electrical stimulation may cause disruption of neuronal firing for either inhibition or stimulation. The inhibition function is similar to creating a lesion to the desired area resulting in permanent inhibition (Johnson et al., 2013). The benefit of DBS is that the inhibition is not permanent and frequency changes allow for variation in inhibition of the structure (Khabarova et al., 2018). Other potential mechanisms include an informational lesion, neurochemical effects such as increased extracellular adenosine or dopamine, disruption of pathological oscillations (especially beta at 12–30 Hz in Parkinson’s disease), desynchronization, or shifting of resonant frequency, alterations in synaptic plasticity by long-term potentiation or other mechanisms, large-scale network reorganization, or neuroprotection/neurogenesis. Mechanisms are likely to be different for different effects based on the timing of therapeutic benefit after application of stimulation: tremor responds in seconds, rigidity in minutes, axial Parkinson’s symptoms in hours, and dystonia after several months.

## Physiology of Blood Pressure and the Pathogenesis of Hypertension

The regulation of blood pressure is a complex process that involves the interplay of a many organ systems, with contributions from the central nervous system (CNS), autonomic nervous system, kidneys, heart, and vasculature (Khor and Cai, 2017). A simplified model of blood pressure homeostasis consists of modulation of the autonomic nervous system, renin-angiotensin-aldosterone system (RAAS), and local endothelial control. The interplay between the autonomic nervous system and RAAS is significant for allowing increased accuracy and flexibility in regulation of organ perfusion. Mean arterial pressure is based on cardiac output and total peripheral resistance (TPR). Cardiac output can be modulated by either altering the contractility of the myocardium or by changing preload, as well as increases in heart rate. Sympathetic outflow increases both myocardial contractility and heart rate via b1-adrenergic receptors. Sympathetic activity leads to an increase in TPR, notably via vasoconstriction (Raven and Chapleau, 2014). On the other hand, the parasympathetic nervous system acts to decrease heart rate. The RAAS uses juxtaglomerular cells to sense a decrease in blood flow through the afferent renal arteriole. Renin is released into the circulation, which then acts upon angiotensinogen, and converts it to angiotensin 1. Angiotensin converting enzyme (ACE), a common target of hypertensive drugs, converts angiotensin 1 to angiotensin 2. Angiotensin 2 causes vasoconstriction as well as the release of aldosterone. Aldosterone, in turn, causes salt and water retention. Thus, both effector actions lead to an increase in blood pressure. The baroreceptor reflex monitors arterial pressure at the carotid sinus and coordinates an appropriate response via modulation of sympathetic and parasympathetic tone (Eckberg and Orshan, 1977; Khor and Cai, 2017). When blood pressure is increased, natriuresis is promoted by natriuretic peptides and by the kinin-kallikrein system (Khor and Cai, 2017). It should also be noted that vascular tone can be regulated by factors produced locally by endothelial cells (Khor and Cai, 2017).

## Central Nervous System Control of Blood Pressure

The central autonomic network (CAN) is the CNS integration circuit of autonomic information from the periphery and functions to effect an appropriate autonomic response to the environment (Hyam et al., 2012). Input to this network consists of peripheral autonomic information, both parasympathetic and sympathetic, relayed to the nucleus tractus solitarius (NTS) (Hyam et al., 2012). Information in the NTS is then sent rostrally to areas including the hypothalamus and insula (Hyam et al., 2012). Parasympathetic outflow is sent to the nucleus ambiguus and dorsal motor nucleus of the vagus, and sympathetic outflow occurs through the rostral ventrolateral medulla via the spinal intermediolateral cell column (Hyam et al., 2012).

The CAN also contains higher structures that have the capability to alter autonomic activity without input from the periphery, such as in instances where an increase in activity is solely imagined (Hyam et al., 2012). The cortical structure involved in this anticipatory process is thought to be the anterior cingulate (Hyam et al., 2012). Subcortical structures, such as the thalamus, subthalamic nucleus (STN), and periaqueductal gray (PAG), are also implicated in the higher-center modulation of the CAN (Hyam et al., 2012).

## Hypothalamus in the Pathogenesis of Hypertension

The hypothalamus plays an important role in the pathogenesis of hypertension (Khor and Cai, 2017). Antidiuretic hormone (ADH), glucocorticoids, mineralocorticoids, alpha-melanocyte stimulating hormone (α-MSH), and leptin are all thought to be involved in this intricate process (Khor and Cai, 2017). Increased ADH release results in peripheral vasoconstriction, RAAS activation, and increased sympathetic output from the hypothalamus itself via an autocrine function (Khor and Cai, 2017). Cortisol activity on MRs in the hypothalamus results in increased sympathetic output and RAAS activation (Khor and Cai, 2017). Leptin acts on arcuate nucleus of the hypothalamus, dorsomedial hypothalamus, and the ventromedial hypothalamus (Khor and Cai, 2017). Inhibition of leptin signaling through destruction or inhibition of the leptin receptors led to decreased blood pressures in mouse models (Simonds et al., 2014; Khor and Cai, 2017). α-MSH is produced in the hypothalamic arcuate nucleus (Khor and Cai, 2017). Interestingly, when a-MSH binds to the melanocortin receptor 4 (MC4) in the hypothalamus in an autocrine fashion, an increase in sympathetic outflow results (Khor and Cai, 2017).

## Salt-Sensitivity

Salt-sensitive changes in sympathetic outflow are mediated by the paraventricular nucleus of the hypothalamus acutely, and by the forebrain hypothalamus chronically (Carmichael and Wainford, 2015). Hypertension in humans is often related to salt regulation by the kidneys since the kidneys manage body fluid concentrations and homeostasis mainly by monitoring sodium content and excreting or retaining sodium as needed (Elijovich et al., 2016). Salt sensitivity is a quantitative trait inherent to populations including African Americans, older adults, and patients with comorbid conditions such chronic kidney disease, diabetes mellitus, or metabolic syndrome (Elijovich et al., 2016). Patients with salt sensitivity experience increased blood pressures in response to increased sodium intake. 51% of hypertensive and 26% of non-hypertensive patients experience salt sensitivity and have increased risk of cardiovascular events (Elijovich et al., 2016).

The exact mechanism of salt sensitivity is largely unknown. Genetic studies reveal that sodium-bicarbonate cotransporters, SLC4A4, and SLC4A5, as well as striatin mediate salt sensitivity (Guo et al., 2015; Elijovich et al., 2016). Recent studies indicate that salt sensitivity may be more closely related to endothelial damage and the storage of salt rather than kidney malfunction (Choi et al., 2015). It is also possible that mineralocorticoids act on the CNS to increase salt appetite, sympathetic drive, and vasopressin release leading to increased BP (Oki et al., 2012). The MR plays a synergistic role with the paraventricular nucleus of the hypothalamus that is, similarly, involved in BP regulation via sympathetic drive (Oki et al., 2012). MRs in the choroid plexus, hippocampus, hypothalamic nuclei, amygdala, and brain stems play a role in BP medication (Huang et al., 2006; Oki et al., 2012). Central blockade of the receptors, specifically in the hypothalamus, result in decreased sympathetic tone, congestive heart failure, and renal dysfunction through decreased BP in rats (Francis et al., 2003; Felder, 2010). Furthermore, the suprachiasmatic nucleus of the hypothalamus is involved in circadian control, and dysfunction of circadian flavoproteins might be implicated in salt-sensitive hypertension, due to an increase in 3B-HSD levels, which leads to elevated levels of aldosterone (Khor and Cai, 2017). In theory, if DBS were to alter signaling, these same sites could prove to be targets for reducing BP in these patients.

## Arcuate Nucleus

The arcuate nucleus of the hypothalamus plays a significant role in the regulation of sympathetic outflow (Rahmouni, 2016). The arcuate nucleus is located adjacent to the median eminence, a circumventricular organ that can sense circulating factors (Rahmouni, 2016). Outflow from the arcuate nucleus is both to other hypothalamic areas, and to other CNS structures (Rahmouni, 2016). Brainstem structures that receive input from the arcuate nucleus of the hypothalamus include the nucleus ambiguous, NTS, and RVLM, and the interomediolateral cell column of the spinal cord also receives input (Rahmouni, 2016). As the RVLM is a mediator of sympathetic outflow (Hyam et al., 2012), the arcuate nucleus may eventually be seen as a site for BP modulation in hypertensive patients.

## Potential Hypothalamic Alterations Leading to Hypertension

### Inflammation

Inflammation, from circulation or prostaglandin E2 made locally by perivascular macrophages, can activate the periventricular nucleus of the hypothalamus, and cause an increase in sympathetic outflow and ACTH release (Khor and Cai, 2017). Further, reactive oxygen species in the periventricular nucleus modulate sympathetic outflow and hypertension (Khor and Cai, 2017). NF-kB blockade in the periventricular nucleus can reduce angiotensin II-induced hypertension (Carmichael and Wainford, 2015; Khor and Cai, 2017). On the other hand, TNF-a, via the production of PGE2, can drive the periventricular nucleus of the hypothalamus to drive the sympathetic nervous system (Khor and Cai, 2017). Predictably, blockade of TNF-a in the periventricular nucleus reduced angiotensin II-mediated hypertension (Carmichael and Wainford, 2015). Thus, the evidence points towards the periventricular nucleus as playing a role in the development of hypertension with inflammation.

### Leptin Signaling

Leptin levels are increased in obese patients and have been correlated with increased BP (Simonds et al., 2014). Leptin effects and receptors are found mainly in the dorsomedial hypothalamus. Animal models with loss of function leptin mutations or decreased leptin receptors in the dorsomedial hypothalamus demonstrate low BP despite severe obesity (Simonds et al., 2014). Circulating leptin hormone released by adipocytes binds to the leptin receptors in the brain resulting in increased sympathetic activity, increased BP, and increased heart rate putting the patient at risk for cardiovascular events (Simonds et al., 2014). Leptin not only acts at the dorsomedial hypothalamus, but also at the arcuate nucleus of the hypothalamus causing the expression of pro-opiomelanocortin that binds to neurons to stimulate the release of γ melanocyte-stimulating hormones. The melanocyte stimulating hormones in turn act on melanocortin 4-receptors in the paraventricular nucleus of the hypothalamus resulting in increased sympathetic tone and increased BP (Simonds et al., 2014). Studies in mice have also shown that leptin activity in the ventromedial hypothalamus increases sympathetic outflow (Khor and Cai, 2017). This information further demonstrates that the nuclei of the hypothalamus could be potential targets for neuromodulation, and in this case perhaps for refractory hypertension in overweight patients with increased leptin levels.

### History of Blood Pressure Modulation via Electrical Stimulation

Electrical stimulation of a variety of targets has caused changes in blood pressure in both human and animal models. In 1894, it was reported that changes in autonomic output, such as an elevation in blood pressure, were observed when the orbital surface of the frontal lobe was stimulated in animals (Chapman et al., 1949). In the 1930s, this effect was replicated by stimulation of various cortical structures in cats (Hoff and Green, 1936). In 1949 and 1952, experiments showed the cortex of the insula caused blood pressure variation when electrically stimulated in monkeys (Kaada et al., 1949; Hoffman and Rasmussen, 1953). In 1949, electrical stimulation of the orbital surface of the frontal lobe in humans was first described to cause an increase in blood pressure, along with respiratory changes (Chapman et al., 1949). This same group also noted that patients who underwent frontal lobotomy (a permanent lesion) did not develop changes in blood pressure (Chapman et al., 1949). Bilateral stimulation of the rostral cingulate gyri was also shown to cause changes to blood pressure, as well as heart rate and respiratory rate in humans (Pool and Ransohoff, 1949).

## Potential DBS Targets to Reduce Blood Pressure

### Periaqueductal Gray

Within the PAG, there are four functional columns (Green et al., 2010; Hyam et al., 2012). Stimulation of the ventrolateral column causes a decrease in systemic arterial pressure and a decrease in heart rate- thus desired effects in the treatment of hypertension, while stimulation of the dorsomedial and dorsolateral columns causes an increase in systemic arterial pressure and an increase in heart rate (Green et al., 2010; Hyam et al., 2012). It has also been suggested that stimulation of the PAG/PVG can cause a decrease in vascular tone (Carter et al., 2011), which would certainly prove to be beneficial in the treatment of hypertension. The ventrolateral column of the PAG has extensive connections with various other neural structures (O’Callaghan et al., 2014). For instance, it receives afferent input from higher centers such as the lateral hypothalamus and the median preoptic nucleus (O’Callaghan et al., 2014), as well as cortical structures such as the orbital cortex and the infralimbic cortex (O’Callaghan et al., 2014). It supplies efferent output to the rostral ventrolateral medulla, NTS, and the interomediolateral cell column, among others (O’Callaghan et al., 2014). Of note, the RVLM is a site of sympathetic output (Hyam et al., 2012), and thus, this would be a key pathway to interrupt in the treatment of HTN.

Green et al. (2005, 2007, 2010) has reported several instances in which DBS of the PAG caused a change in blood pressure. In 2005, the group used PAG DBS to treat patients with chronic pain. They found that 10 Hz stimulation of the ventral PAG caused a reduction in blood pressure of 14.2 mm Hg systolic and 4.9 mm Hg diastolic, with SBP reduced from 143 to 128 mm Hg when stimulation was applied. They reported the opposite effect on blood pressure when the dorsal PAG was stimulated (Green et al., 2005). They postulated that the effect was due to increased sympathetic outflow, because an increase in TPR and an increase in cardiac contractility were seen (Green et al., 2005). To support this, no change in heart rate was observed in the patients with a reduced blood pressure following ventral PAG stimulation (Green et al., 2005). In 2007, the same group repeated their findings, but this time in a hypertensive patient, who was undergoing DBS for pain control (Green et al., 2007). 2 V stimulation at 30 Hz caused his baseline arterial pressure of 157.4/87.6 mm Hg to fall to 132.4/79.2 mm Hg (Green et al., 2007). This patient had not discontinued his antihypertensive medications at the time of the procedure (Green et al., 2007). In 2010, the same group used heart rate variability as a surrogate for autonomic output (Green et al., 2010) (since high frequency heart rate variability is indicative of parasympathetic activity, and low frequency heart rate variability reflects sympathetic activity (Lacuey et al., 2017) and found that the autonomic nervous system function could be influenced by PAG stimulation, and that this effect might be mediated by both parasympathetic and sympathetic components (Green et al., 2010). Six patients, one with hypertension, were investigated (Green et al., 2010). Electrode placement was 3 mm lateral to the aqueduct between the red nucleus and superior colliculus, less than 10 mm below the AC-PC line (Green et al., 2010).

Lasting changes in blood pressure have been reported with PAG D/BS (Pereira et al., 2010; Patel et al., 2011; Hyam et al., 2012 ), although the reduction in blood pressure at one year might have been confounded by the analgesic benefit of the procedure (Pereira et al., 2010; Hyam et al., 2012). One case report described a lasting response (Patel et al., 2011; Hyam et al., 2012) in a patient who underwent PAG stimulation for a central pain syndrome involving the left hemibody whose pain returned to baseline 4 months after the surgery, but at 27 months, when DBS stimulation was turned off, blood pressure rose by 18/5 mm Hg, indicating a continued effect of DBS (Patel et al., 2011).

Further evidence for DBS of the ventral PAG in treating hypertension was described in 2017 by O’Callaghan et al. (2017). This group treated a patient who had previously tried a variety of medications as well as a baroreceptor activation device to control her blood pressure (O’Callaghan et al., 2017). Over a period of 3 weeks prior to surgery, she had recorded her average blood pressure to be 280/166 mm Hg (O’Callaghan et al., 2017). After 6 months of chronic low-frequency DBS of the vPAG at 4.3 mA, 10 Hz, and a pulse-width of 150 ms, her blood pressure readings of approximately 210/130 mm Hg in the mornings and approximately 230/130 mm Hg in the evenings (O’Callaghan et al., 2017).

### Rostral Subcallosal Neocortex (Brodmann Area 25)

The rostral suballosal neocortex has reciprocal connections with the ventral PAG/PVG and the lateral hypothalamic nuclei as well as many lower centers, including the rostral ventrolateral medulla that is speculated to be the effector location at which sympathetic outflow was decreased in the efferent component of the baroreceptor reflex (Lacuey et al., 2017). Interrupting this sympathetic outflow, from the RVLM, would logically be beneficial in the treatment of hypertension. Stimulation of the rostral subcallosal neocortex was shown to cause a reduction in systolic arterial pressure in four of 12 patients subjected to invasive electroencephalography for epilepsy surgery evaluation (Lacuey et al., 2017). Bipolar and monopolar stimulation was used with a frequency of 50 Hz, a pulse width of 0.2 ms, and train durations lasting as long as 30 s. The intensity began at 1 mA and went no higher than 10 mA. Interestingly, decreases in diastolic arterial pressure or heart rate were not observed, despite the fall in systolic arterial pressure. The resulting decrease in pulse pressure led researchers to believe that the findings were due to a decrease in cardiac contractility that resulted in a lower stroke volume. Further, the absence of a reduction in diastolic pressure lead to the conclusion that the effect was not mediated by alterations in vascular resistance (Lacuey et al., 2017). Furthermore, an added benefit of this target, is that it is a cortical target, as opposed to a deep brain structure, which allows for less invasive access.

### Subthalamic Nucleus

Patients with Parkinson’s Disease who underwent stimulation of the STN with a frequency of over 100 Hz were reported to have a modest increase in heart rate and blood pressure (5 bpm and 5 mm Hg, respectively) (Hyam et al., 2012). The STN has also demonstrated mixed results as a target for improving orthostatic hypotension in patients with Parkinson’s Disease (Hyam et al., 2012). One study showed the baroreceptor sensitivity was maintained along with arterial blood pressure when PD patients were subjected to a head up-tilt test (Hyam et al., 2012). Another study demonstrated that DBS targeting the GPi compared to the STN were comparable in terms of motor outcome, but were not clinically equivalent (Weaver et al., 2012). Due to the clinical success of DBS on the STN for Parkinson’s disease, most research focuses on outcomes within that Parkinson’s population. However, the success in autonomic regulation suggests a possible target for refractory hypertension. The STN is one of the higher neural structures in the CAN (Hyam et al., 2012), and perhaps this is the reason why stimulation of the STN has the aforementioned effects.

## Other Potential Targets

### Posterior Hypothalamus

Found in the diencephalic area of the brain and responsible for homeostatic and vegetative functions, the posterior hypothalamus regulates neurotransmitter release connecting the amygdala, hippocampus, and retina (Messina et al., 2016). Stimulation of the posterior hypothalamus was demonstrated in one patient to facilitate the maintenance of SBP during head up-tilt testing (Hyam et al., 2012), TPR and DBP also increased during this test (Hyam et al., 2012). Posterior hypothalamus stimulation has been implicated in cluster headache treatment through trigeminal autonomic cephalgias that regulates autonomic symptoms such as lacrimation and rhinorrhea (Messina et al., 2016). Specifically, posterior hypothalamus stimulation in cats and monkeys lead to decreased carotid resistance that caused increased facial temperature (Messina et al., 2016). However, this mechanism of reduced carotid resistance could be harnessed to control systemic blood pressure in refractory patients.

### Rostrocaudal Cingulate Gyrus

Systolic and diastolic arterial pressure rose in 8 of 12 patients who received bilateral stimulation of the rostrocaudal cingulate gyrus (in 1949), and one patient had a reduction in these pressure (Pool and Ransohoff, 1949; Lacuey et al., 2017). This group also noted changes in heart and respiratory rate, and thus theorized that the rostral cingulate gyrus exerted some autonomic control (Pool and Ransohoff, 1949).

### Orbitofrontal Gyrus

In 1949, 6 of 9 patients had an increase in blood pressure when the orbital surface of the frontal lobes was electrically stimulated (Hariz, 2014; Riva-Posse et al., 2014). Recently, orbitofrontal gyrus stimulation has shown success in treating OCD through recurrent inhibition of neurons (McCracken and Grace, 2007). This pathway has largely been associated with the reward pathway specifically in treating refractory depression with anhedonia (Schlaepfer et al., 2008). The orbitofrontal cortex has also been implicated in the integration of sensory and autonomic signals to modulate behavior that could be implicated in blood pressure responses (Kringelbach and Rolls, 2004; Kringelbach, 2005).

### Insular Cortex

The insular cortex has been found to have varied effects when stimulated in human subjects. Oppenheimer et al. (1992, 1996) claims that right insular cortex stimulation leads to an increase in heart rate and blood pressure, while left insular cortex stimulation causes bradycardia (). This same group postulates that as the insular cortex is an area of autonomic and limbic convergence, that it may be responsible for changes in cardiac function with emotional stress (Oppenheimer et al., 1996).

## Conclusion

The homeostatic mechanisms that function to maintain blood pressure are complex and intimately related to the CNS. Because the brain is involved in control of these processes, neuromodulation of structures offer promise in the treatment of hypertension. The most thoroughly studied target is the periaqueductal gray, although other areas such as the rostral subcallosal neocortex and insular cortex have also been described. If chronic modulation of these structures is able to produce sustained normalization of blood pressure, DBS might become a treatment modality for patients who do not otherwise respond to antihypertensive therapy.

## Author Contributions

JM designed and directed the project. JM and TO provided the technical expertise. RE and AG underwent literature review and wrote the paper with input from all authors.

## Conflict of Interest Statement

The authors declare that the research was conducted in the absence of any commercial or financial relationships that could be construed as a potential conflict of interest.
